# Expanding the clinical spectrum of *PPP3CA* variants - alternative isoforms matter

**DOI:** 10.1186/s13023-024-03507-0

**Published:** 2024-12-20

**Authors:** Silvia Castiglioni, Laura Pezzoli, Lidia Pezzani, Antonella Lettieri, Elisabetta Di Fede, Anna Cereda, Silvia Ancona, Andrea Gallina, Elisa Adele Colombo, Chiara Parodi, Paolo Grazioli, Esi Taci, Donatella Milani, Maria Iascone, Valentina Massa, Cristina Gervasini

**Affiliations:** 1 Department of Health Sciences, Università degli Studi di Milano, Milan, Italy; 2 Medical Genetics Laboratory, Papa Giovanni XXIII Hospital, Bergamo, Italy; 3 Pediatrics, Papa Giovanni XXIII Hospital, Bergamo, Italy; 4 Fondazione IRCCS Ca’ Granda Ospedale Maggiore Policlinico, Milan, Italy; 5 “Aldo Ravelli” Center for Neurotechnology and Experimental Brain Therapeutics, Università degli Studi di Milano, Milan, Italy

**Keywords:** PPP3CA, calcineurin, DEE-91, LCLs, UPS

## Abstract

**Background:**

the protein phosphatase 3 catalytic subunit alpha (*PPP3CA*) gene encodes for the alpha isoform of the calcineurin catalytic subunit, which controls the phosphorylation status of many targets. Currently, 23 pathogenic variants of PPP3CA are known, with clinical manifestations varying by mutation type and domain.

**Results:**

through whole exome sequencing, we found two *de novo* variants in *PPP3CA*: a frameshift variant predicted leading to a truncated protein in Pt.1 and a splicing variant in Pt.2 associated with mild phenotype. PPP3CA is ubiquitously expressed with tissue-specificity of; namely, splicing isoform 1 prevailing over isoform 2 in the central nervous system. By analyzing isoform distribution in patient-derived cell lines, we highlight a skewed expression of both isoforms in Pt.1, whereas only isoform 2 shows a moderate reduction in Pt.2. In contrast, we did not observe significant abundance changes at the protein level. Cell lines derived from Pt.1 showed a reduced proliferation, associated with an increase in cell death and the upregulation of the unfolded protein response (UPR) pathway.

**Conclusion:**

data suggest that an aberrant PPP3CA protein in Pt.1 could lead to UPR activation resulting in increased cell death. In Pt.2 an imbalance between the two main isoforms possibly explains the peculiar pathological manifestations, such as a moderate developmental delay.

## Introduction

The protein phosphatase 3 catalytic subunit alpha isoform (*PPP3CA*) is a gene located on chromosome 4q24 that encodes for the 61-kD alpha isoenzyme of the Calcineurin A (CnA). CnA forms a heterodimer with a regulatory subunit, Calcineurin B (CnB), to produce calcineurin (CaN), a Ca^2+^/calmodulin-regulated protein phosphatase. CaN is ubiquitously expressed, with the highest levels found in brain [1] and is conserved from unicellular organisms to *Homo sapiens* [2]. CaN controls the phosphorylation state of many targets including important synaptic receptors, transcription factors, ion channels and cytoskeleton proteins [2–5].

**Fig. 1:**
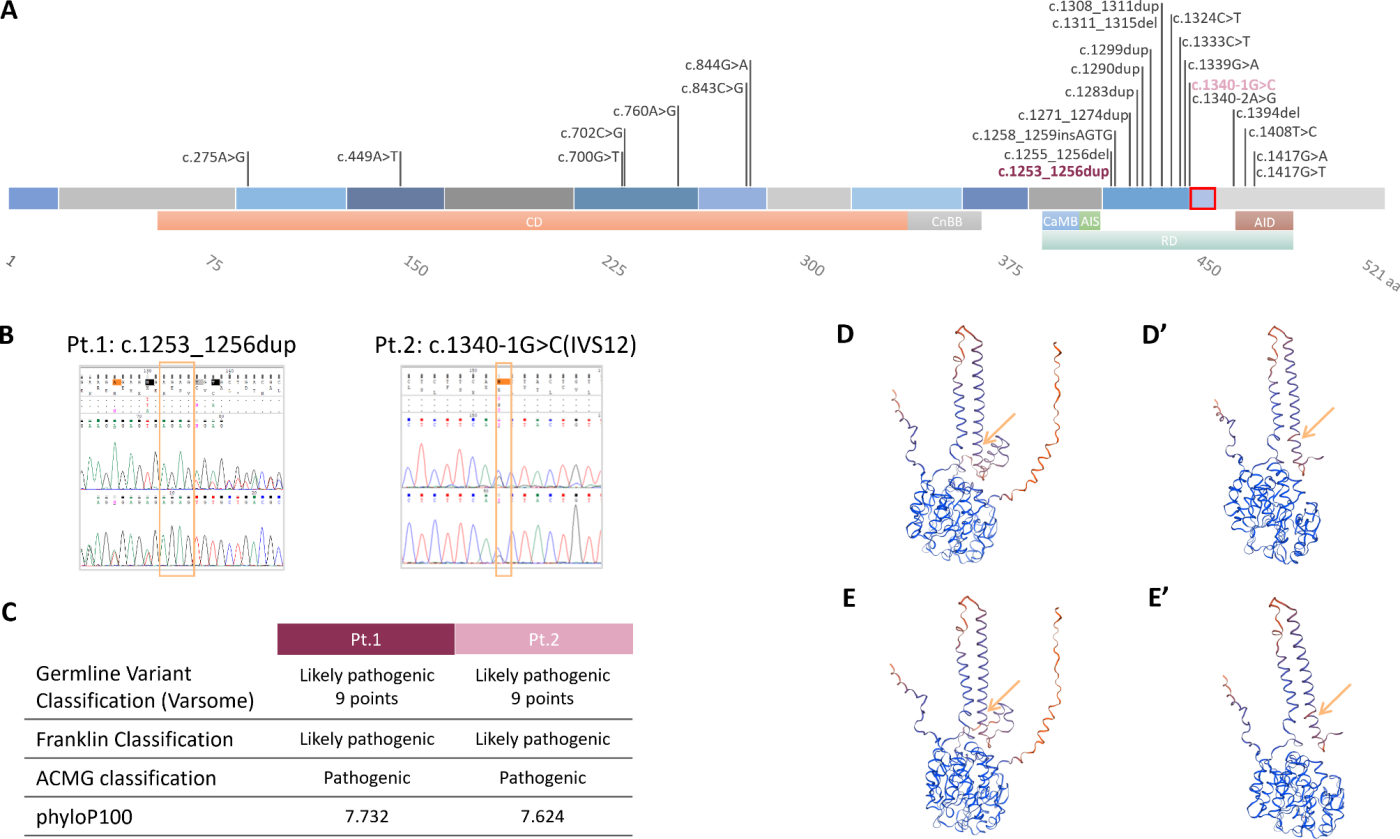
Schematic representation of PPP3CA pathogenetic variants and characterization of the two under investigation. (**A**) Variants described so far in *PPP3CA* with the protein domains represented below (CD = catalytic domain, CnBB = calcineurin B binding site, RD = regulatory domain, CaMB = calmodulin binding site, AIS = autoinhibitory segment, AID = autoinhibitory domain). In plum-colored and pink are the variants of Pt.1 and Pt.2, respectively. Exon 13 is squared in red, which is present in isoform 1 (canonical) and absent in isoform 2. (**B**) Electropherogram confirmation by Sanger sequencing on DNA of the variants found in the two patients; the variant is reframed in yellow. (**C**) Pathogenicity score of patients’ variants using different prediction software. (**D**-D’) Folding prediction of isoform 1 protein folding wt and mutated in Pt.1 (Swiss-model). (**E**-E’) Prediction of isoform 2 protein folding wt and mutated in Pt.1 (Swiss-model). The yellow arrow indicates the starting frameshift amino acid

PPP3CA has several domains, from the N-terminus: a catalytic domain (CD), a calcineurin B binding site (CnBB), a regulatory domain (RD) including a calmodulin binding site (CaMB), an autoinhibitory segment (AIS) and an autoinhibitory domain (AID) (Fig. 1A) (www.uniprot.org) [6].

Five different isoforms have been described for *PPP3CA*: Q08209-1 521 aa (isoform 1), that has been chosen as the canonical one; Q08209-2, 511 aa (isoform 2); Q08209-3, 469 aa (isoform 3); Q08209-4, 289 aa (isoform 4); Q08209-5, 454 aa (isoform 5) (www.uniprot.org) [7]. The most abundant are isoform 1 and isoform 2, which differ for the enclosure of 30-nucleotide-long exon 13 in isoform 1. Their expression is tissue-specific: isoform 1 is highly expressed in the nervous system and its relative abundance seemed to rise during brain development [8].

*PPP3CA de novo* heterozygous variants have been related to three different disorders depending on the mutation type and which domain it falls into. All share a severe clinical outcome and epileptic seizures as a hallmark, which can be attributed to CaN involvement in the regulation of synaptic transmission [6, 8]. Variants affecting CD and with a loss-of-function mechanism lead to developmental and epileptic encephalopathy-91 (DEE91, OMIM #617711). DEE91 is a condition characterized by delayed psychomotor development, severe impaired intellectual development with poor or absent speech, onset of refractory multifocal seizures, absence of independent walking, hypotonia and cortical visual impairment [9]. Missense variants falling into AID are associated to arthrogryposis, cleft palate, craniosynostosis, and impaired intellectual development (ACCIID, OMIM #618265), with a gain-of-function mechanism, also associated with seizures and bone abnormalities [6, 8]. Truncating variants in RD lead to a clinical spectrum, which includes developmental delay, intellectual disability, abnormal electroencephalogram, and epilepsy; the underlying pathogenetic mechanism still need to be elucidated though [6].

To date 24 *PPP3CA* pathogenic variants in 29 patients have been described: 7 variants in CD coding region, 17 variants in RD coding region, with a mutational hot spot at exon 12 involving 11 variants among them [6, 8–12] (Fig. 1A).

Here we describe two patients carrying two variants in *PPP3CA* with a distinct severity of clinical gravity. Furthermore, exploiting lymphoblastoid cell lines derived from patients and controls, we investigated the effect of the two variants at the transcript and protein level, also investigating the involvement of the UPR pathway.

## Methods

### WES analysis and variant validation

The whole exome sequencing (WES) analysis was performed on DNA extracted from the peripheral blood of the proband and its parents. Regions containing exons and genome splicing sites are enriched by solution hybridisation (Agilent, SureSelect Clinical Research Exome) and analysed by massively parallel sequencing (Illumina, PE 2 × 150). The analysis is focused on the disease genes associated with the clinical features of the probands and in the absence of certain familiarity, is performed considering an autosomal recessive or *de novo* dominant. Identified variants are reported according to the HGVS nomenclature (hgvs-nomenclature.org).

Sanger sequencing was used on DNA to confirm the identified variant in Pt.1 (forward primer CGGAGAGCCAAGATAGAGGG, reverse primer CAGGGCTCTAATGACACAGC) and Pt.2 (forward primer TCTGTTTTGTGTGCCA, reverse primer TCAACCCACACTCTGT). Sanger sequencing has also been carried out on both patients cDNA to verify/exclude the presence of aberrant transcripts (forward primer CAGCCCGGAAAGAGGTGATA, reverse primer GTGCCGTTAGTCTCTGAGGT). The two variants have been reported in LOVD website with the variant ID #0000987643 (Pt.1) and ID#0000987644 (Pt.2) (www.lovd.nl).

### Lymphoblastoid cell lines

From the lymphocytes obtained from the peripheral blood of the two patients and from healthy controls, stable lymphoblastoid cell lines (LCLs) were obtained by transformation with Epstein-Barr virus (Gaslini Genetic Bank, Genova, IT) [13]. The Ethics Committee of the Università degli Studi di Milano approved the use of these cell lines (Comitato Etico number 99/20, 17 November 2020). LCLs were cultured with RPMI 1640 medium with L-Glutamine (ECB2000, Euroclone) supplemented with 20% fetal bovin serum (FBS, ECS0180L, Euroclone) and 1% penicillin and streptomycin (ECB3001, Euroclone).

### RNA extraction and RT-qPCR

Total RNA was obtained from LCL cell pellets using the phenol/chloroform method NucleoZOL (740404.200, Macherey-Nagel) and quantified at the Nanodrop spectrophotometer. 1 µg of RNA was then retrotranscribed using All-in-one 5x RT MasterMix kit (G592, Abm) and random primers. RT-qPCR was performed on CFX Opus 96 system thermocycler (Bio-Rad) using Master Mix with TB Green (RR420A, Takara Bio Inc.), specific primers (Table 1) and 2 µl of cDNA diluted 1:10 for each well. The quantification cycle (Cq) value and the ΔΔCq were calculated relative to control samples using Cq threshold values normalized to the housekeeping genes *GAPDH/RPLP0/RPL13A* (Table 1). Experiments were performed in biological and technical triplicate.

**Table 1:** Specific primer sequences used for RT-qPCR experiments

Primer Name	Sequence (5’ -> 3’)
Pr11F	GGAACAAGATCCGAGCAATAGG
Pr12_13R	TAGCCTCAACAGTAGCGCTT
Pr12_14R	CTTTGATAGCGCTTTGCAGGG
BiP_f	TGTTCAACCAATTATCAGCAAACTC [14]
BiP_r	TTCTGCTGTATCCTCTTCACCAGT [14]
CHOP_f	GACCTGCAAGAGGTCCTGTC [14]
CHOP_r	CTCCTCCTCAGTCAGCCAAG [14]
XPB1sp_f	GGAGTTAAGACAGCGCTTGG [14]
XPB1sp_r	GCCTGCACCTGCTGCGGA [14]
GAPDH_f	AGCCACATCGCTCAGACAC [15]
GAPDH_r	GCCCAATACGACCAAATCC [15]
RPLP0_f	TCTACAACCCTGAAGTGCTTGAT [15]
RPLP0_r	CAATCTGCAGACAGACACTGG [15]
RPL13A_f	CCTGGAGGAGAAGAGGAAAGAGA [16]
RPL13A_r	TTGAGGACCTCTGTGTATTTGTCAA [16]

### Protein extraction and Western blot analysis

Patients and controls LCLs were lysed in S300 buffer (50 mM HEPES pH 7.6, 300 mM NaCl, 0.1% NP40, 2 mM MgCl2, 10% glycerol) with nuclease and phosphatase protease inhibitor cocktail (P8340, Sigma-Aldrich). Lysates were centrifuged at 10,000 g for 10 min at 4 °C and protein concentration was determined with the Bradford assay (Bio-Rad). Protein lysates were denatured in Laemmli sample buffer 4x (LSB, #1610747, Bio-Rad) supplemented with β-mercaptoethanol and then used for SDS-PAGE separation through 10% Mini-PROTEAN TGX Precast Protein (4561034, Bio-Rad). Proteins were transferred to nitrocellulose membranes (Bio-Rad) in Transfer buffer 1x and immunoblotted with rabbit anti-PPP3CA (1:1000; A300-908 A, Bethyl Laboratories) or rabbit anti-GAPDH (1:1000; 5174 S, Cell Signaling) followed by goat anti-rabbit HRP-conjugated antibodies (1:2000; 12–348, Merck Millipore). Images of the chemiluminescent signal were acquired by the Chemidoc imaging system using Image Lab software (v5.2.1, Bio-Rad) after incubating the membranes with ECL (#1705061 Bio-Rad). For quantification, 3 independent experiments were performed, the OD of the signal was measured with ImageJ software (v1.52a, NIH) and the mean pixel intensity was calculated.

### MTT assay

To assess the proliferative capacity of each LCLs, the MTT (3-[4,5-dimethylthiazol-2-yl]-2,5 diphenyl tetrazolium bromide) assay was performed for 4 consecutive days, starting from day 0 with a concentration of 250,000 cells/ml, taking 1 ml each day from a different flask and adding MTT stock solution (5 mg/ml MTT powder in sterile white serum-free medium) (M2003, Sigma-Aldrich) leading to the formation of a purple precipitate. MTT is a salt that can cross the cell membrane and mitochondrial membranes, where it is converted to formazan in metabolically active cells. After a two-hour incubation, the supernatant was discarded and isopropanol was added, which allows lysis of the cells. The reaction is chromogenic, and the amount of formazan produced can be measured by colorimetric analysis at 590 nm on a microplate spectrophotometer (Ensight PerkinElmer).

### Cytofluorimetry

To assess apoptosis, a pellet of 4 million LCLs from patients and controls was prepared using CytoFix/Perm Kit (554714, BD Biosciences). This kit includes a solution to fix cells (fixation solution), a solution to permeabilize cells (permeabilization solution), and a washing solution (BD Perm/Wash™ Buffer). Subsequently, the pellets were labeled with the antibody anti-active Cas3 APC-conjugated (560626, BD Pharmingen). In parallel, tubes with normal mouse IgG isotype control APC-conjugated and one tube with no antibody for negative control were prepared. Tubes were read at BD FACSVerse™ flow cytometer, data acquisition and analysis were done BD FACSuite v1.0.6 software. Experiments were performed in biological and technical triplicate.

### Statistical analysis

Data were analyzed using Excel and expressed as mean ± Standard Error of the Mean (SEM). Statistical analysis of the experimental data was performed using the *Student t-test*, considering significance for p value < 0.05 (* *p* < 0.05; ** *p* < 0.01; *** *p* < 0.001; **** *p* < 0.0001).

## Results

### Clinical report

#### Patient 1

The proband (Pt.1) was the only son of a healthy non-consanguineous Italian couple, born from uneventful pregnancy. By the age of two months, he had myoclonic seizures requiring hospitalization, and unresponsive to multiple anti-epileptic treatments. Preferential head posture turned to the right, absence of eye contact, erratic conjugate eye movements; fluctuating hypertonia of the four limbs, and poor active motility were noted. No facial dysmorphisms or other minor anomalies were associated. Head Magnetic Resonance only showed thinned corpus callosum. In the first years of age he showed progressive respiratory failure needing oxygen supplementation, dysphagia, tetra-paresis, severe alteration of the sleep-wake rhythm, and refractory polymorphous seizures of variable intensity and duration both in wakefulness and asleep. Percutaneous endoscopic gastrostomy (PEG) was placed. Polysomnography documented a markedly disorganized and poor electroencephalography (EEG) with numerous episodes of a possible critical nature. (Table 2). The proband died at the age of 7 years due to respiratory complications.

#### Patient 2

Pt.2 The proband (Pt.2) was the second-born to a healthy non-consanguineous Italian couple, born from uneventful pregnancy. At birth a double renal district has been detected and followed up in the first months of age. He showed regular growth. Language delay was noted from the age of 2 years, with initial diagnosis of autism spectrum disorder, for which he started Applied Behavior Analysis (ABA) rehabilitation. When he was 5 years old, he had a first critical episode of epilepsy; during hospitalization 3 new episodes of generalized tonic-clonic seizures with desaturation have been documented. EEG showed sporadic frontal and diffuse paroxysmal anomalies. Head MRI (magnetic resonance imaging) was normal. Valproic acid therapy was started with benefit. He also had moderate developmental delay, and emotional regulation disorder. At the age of 8 years, no more seizures have been reported and the patient showed improved speech with dyslalia, good strength and coordination, difficulties in relationships. He attends primary school with support. EEG shows marked improvement, with only slow activity excess (Table 2). To date, this patient is the most different from all those described, with a milder phenotype based on developmental, respiratory and neurological symptoms.

**Table 2:** Clinical table of the main two patients’ clinical features. EEG: electroencephalography, PEG: Percutaneous Endoscopic Gastrostomy, GE reflux: Gastroesophageal reflux

	Pt.1	Pt.2
Ethnicity	Caucasian	Caucasian
Sex	Male	Male
Year of birth/death	2017 − 2014	2015-still alive
Pathogenic variant	c.1253_1256dup (p.Ser419ArgfsTer33)	c.1340-1G > C (IVS12)
Prenatal period	Normal	Normal
Gestation	38w + 4d	37w + 5d
Development delay	Severe	Moderate
Muscle tone	Fluctuating in the limbs	Normal
Autistic Features	Not evaluable	Moderate
Age of seizure onset	2 months	4 and half years
When	Wakefulness, fall asleep, sleep	Wakefulness, sleep
Seizure Types	Polymorphic (myoclonic, focal, tonic)	Tonic-clonic
Therapy	Not effective polytherapy	Valproic acid
EEG	Markedly disorganized	Discreetly organized
Dysmorphic Features	Absence	Absence
Skeletal Abnormalities	Absence	Absence
Sleep-wake rhythm	Severe alteration	Normal
Respiratory anomalies	Respiratory failure, oxygen dependence	Absence
Feeding problems	PEG with ketogenic diet, GE reflux	Absence
Vision problems	Incorrect conjugated eye movements, absence of eye contact	Absence
Speech	Absence	Moderate delay, dyslalia
Deambulation	Absence	Normal
Other findings	Tetraparesis	Absence

### Genetics

We performed trio-based whole-exome sequencing (WES-trio) on the two probands and their parents. After filtering, in both patients we identified a *de novo* heterozygous variant in the *PPP3CA* gene, a variant already reported in literature [17].

In Pt.1 we identified the c.1253_1256dup variant at position Chr4(GRCh38):g.101,032,350 (NM_000944.5:c.1253_1256dup - p.(Ser419Argfs*33)), which causes a frameshift at the beginning of exon 12 with the formation of a premature termination codon (PTC) (Fig. 1A) (Table 3).

In Pt.2 we identified the c.1340-1G > C variant at position Chr4(GRCh38):g.101,029,196 (NM_000944.4:c.1340-1G > C), which alters the splice acceptor site of intron 12 of *PPP3CA* (Fig. 1A).

WES results were confirmed by Sanger sequencing using specific primers (Fig. 1B) on DNA and cDNA of patients LCLs, although for Pt.2 the variant is not appreciable in cDNA; additionally, was ruled out the presence of aberrant transcripts caused by the Pt.2 splicing variant.

The two variants were predicted to be likely pathogenic or pathogenic by different software (Varsome, Franklin, ACMG classification) (www.varsome.com, www.franklin.genoox.com) and alignment sites were found to be evolutionarily conserved (phylop100) (Fig. 1C).

In silico analysis of Pt.1 variant predicted a premature stop codon in exon 13 at position 450 for isoform 1 (Fig. 1D-D’), while for isoform 2 the premature termination codon is predicted to be in exon 14 at position 456 (Fig. 1E-E’) (expasy.org).

**Table 3:** Wildtype (wt) and aberrant (mut) aminoacid sequences due to Pt.1 frameshift variant starting with exon 12. The amino acid fragment coding by exon 13 is written in green; new amino acids caused by frameshift reading are underlined (expasy.org)

	Amino acid sequence	aa
Iso1 wt	…REESESVLTLKGLTPTGMLPSGVLSGGKQTLQSATVEAIEADEAIKGFSPQHKITSFEEAKGLDRINERMPPRRDAMPSDANLNSINKALTSETNGTDSNGSNSSNIQ	521
Iso1 mut	…REESE**RECADAERLDPNWHAPQRSTFWREANPAKRYC**	450
Iso2 wt	…REESESVLTLKGLTPTGMLPSGVLSGGKQTLQSAIKGFSPQHKITSFEEAKGLDRINERMPPRRDAMPSDANLNSINKALTSETNGTDSNGSNSSNIQ	511
Iso2 mut	…REESE**RECADAERLDPNWHAPQRSTFWREANPAKRYQRIFTTT**	456

### In vitro studies

#### *PPP3CA* isoforms expression studies

**Fig. 2:**
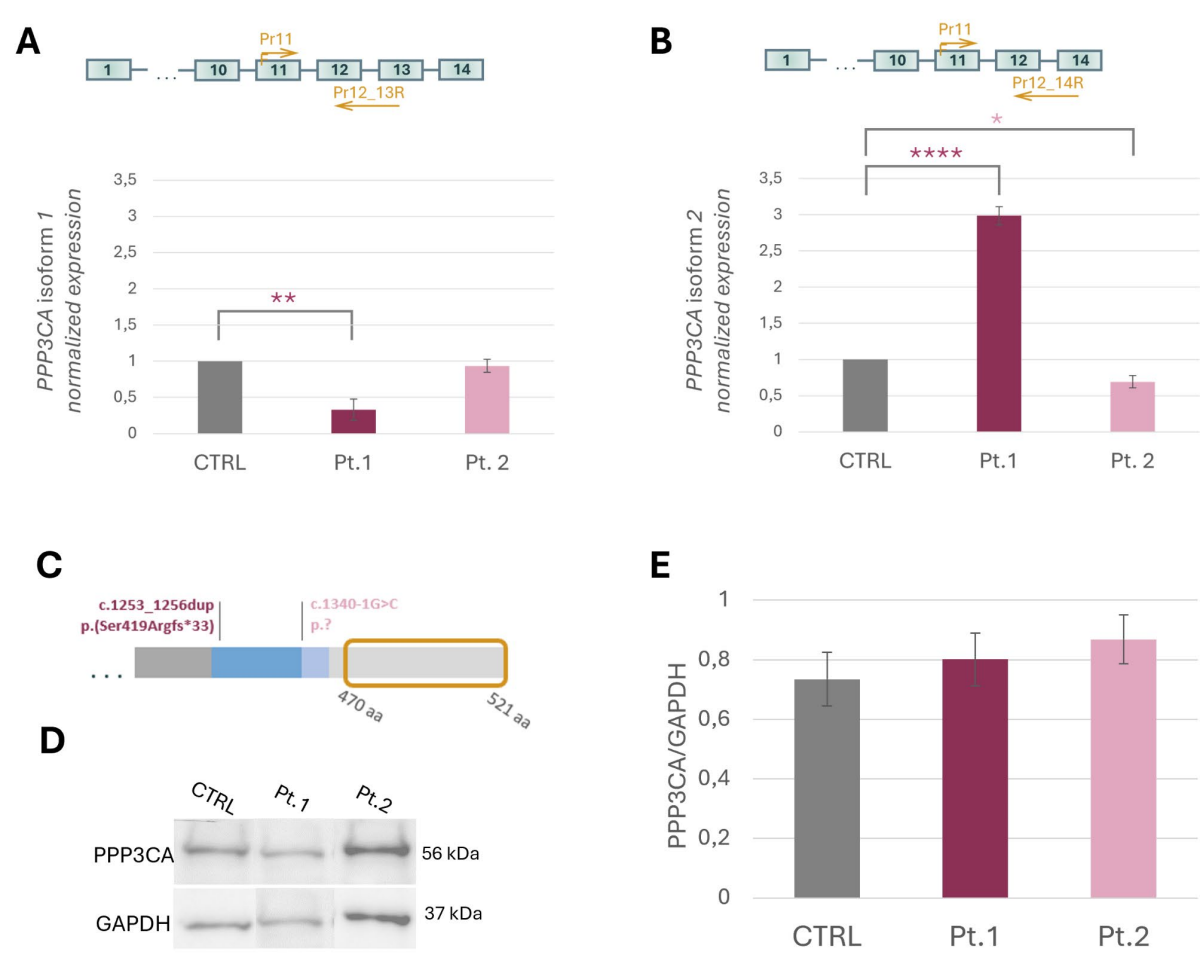
Expression analyses of PPP3CA transcript and protein in patients and controls LCLs- controls pool (grey bar), Pt.1 (plum-colored bar) and Pt.2 (pink bar) (**A**) mRNA relative expression of *PPP3CA* isoform 1 (**B**) mRNA relative expression of *PPP3CA* isoform 2. The yellow arrows indicate the location of the primers used. (**C**) Representation of C-terminal partion of the protein; the yellow box indicates the antibody binding zone; (**D**) western blot analysis of PPP3CA protein (56 kDa) normalized on GAPDH (37 kDa), (**E**) plot presenting the protein quantification. Data analysis was performed using *Student’s t-test* as statistical method, significance was considered for *p* < 0.05 (* *p* < 0.05; ** *p* < 0.01; *** *p* < 0.001; **** *p* < 0.0001)

First, we studied the two major PPP3CA isoforms expression: isoform 1, which retains exon 13, and isoform 2, in which exon 13 is excised by exon skipping. The experiments were performed on cDNA derived from RNA extracted from LCLs derived from the two patients (Pt.1 and Pt.2) and three controls (CTRL). To study the two isoforms separately, we designed specific reverse primers for the different junctions: exon12–exon13 junction for isoform 1 and exon12–exon14 junction for isoform 2; for both amplification we used the same exon 11 forward primer (Fig. 2A-B). Isoform 1 resulted to be under expressed in Pt.1 (*p* = 0.01) with a fold decrease of 0.3 while no changes in its expressions were observed in Pt.2 compared to controls. In stark contrast, isoform 2 was 2.8 more expressed in Pt.1 than in controls (*p* = 9.2E-05), while in Pt.2 this isoform was downregulated (*p* = 0.02) with a significant decrease of 0.7 compared to controls (Fig. 2A-B).

#### PPP3CA protein-level analysis

Having established that the two patients’ pathogenic variants affect the *PPP3CA* two isoforms expression, we evaluated the protein translation by western blot (WB) analysis on LCLs lysates derived both from patients and controls. No statistically significant difference was found in the abundance of PPP3CA in patients compared with controls. (Fig. 2C-D-E).

#### Patients LCLs proliferation and survival

**Fig. 3:**
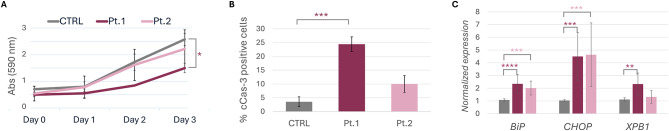
Analysis of cell viability/apoptosis and UPR genes relative expression in PPP3CA patients and controls LCLs. (**A**) Line graph showing the LCLs viability evaluated by MTT assay and (**B**) plot showing the Cleaved Caspase-3 (c-Cas-3) evaluated using cytofluorimetry after 48 h of culture. (**C**) Relative expression of three unfolded protein response genes. Grey line and bar indicate the control pool, the plum-colored line and bar indicate Pt.1 and the pink line and bar indicate Pt.2. Data analysis was performed using *Student’s t-test* as statistical method, significance was considered for *p* < 0.05 (* *p* < 0.05; ** *p* < 0.01; *** *p* < 0.001; **** *p* < 0.0001)

To assess the proliferative capability of the lymphoblastoid cell lines derived from the two patients, the MTT assay was performed for four consecutive days and compared to controls. The proliferation curve of Pt.2 was overlapping with that of controls, while proliferation was significantly reduced in cells derived from Pt.1, as early as the first day of measurement (at day 4, *p* = 0.04) (Fig. 3A).

Given the lower proliferation rate of Pt.1 LCL we investigated the mortality of these cells to assess if the reduced proliferative capacity was associated with increased cell death. Cleaved Caspase 3 (cCas-3) was analyzed by cytofluorimetry after 48 h of culture. Pt.1 had a higher percentage of cCas-3 positive cells than controls, a difference found to be statistically significant (*p* = 0.001) (Fig. 3B). These data suggest that the altered proliferation rate of Pt.1 LCL is a consequence of an increased number of apoptotic cells.

#### UPR genes expression studies

Data reported above about two novel *PPP3CA* variants show altered expression levels of isoform 2 (Pt.2) or both isoform 1 and 2 (Pt.1) at the mRNA level and predict altered protein products at the protein level for Pt.1. In cell lines derived from both patients, a significant depression of proliferation and enhancement of apoptosis was shown.

Among the pervasive regulatory actions exerted by CaN, one revolves around the attenuation of cell stress, and specifically ER stress, by contributing to the mechanisms of calcium homeostasis and by modulating the UPR pathway through the association with, and the dephosphorylation of protein partners such as Calnexin and Perk protein kinase among others.

Hence, we investigated the expression of UPR marker genes (*BiP*, *CHOP*, *XPB1*) in the LCLs derived from patients and controls. In Pt.1 LCLs, all three genes were found to be overexpressed: *BiP* 2.3-fold (*p* = 0.0001), *CHOP* 4.5-fold (*p* = 0.0007) and *XPB1* 2.3-fold (*p* = 0.004), respectively (Fig. 3C). In Pt.2 LCLs, 2 out of 3 genes were found to be overexpressed: *BiP* 2-fold (*p* = 0.0009) and *CHOP* 4,6-fold (*p* = 0.0008), respectively (Fig. 3C). Data, thus, agree with a sustained activation of UPR at the transcriptional level in both patient-derived LCLs.

## Discussion

Calcineurin (Cn) is a protein phosphatase, that regulates by both physical interaction and dephosphorylation an extensive array of protein targets [2–5]. Moreover, CaN has been known to be involved in ER calcium signaling and homeostasis, as well as in modulating the activity of ER-associated UPR mediators such as Calnexin and Perk [18, 19]. UPR is an adaptive cell response to ER stress, which is activated when ER proteostasis is broken with an accumulation of misfolded and unfolded proteins in the ER [20, 21]. A prolonged activation of this pathway can drive the cell to apoptosis [20, 21]. In this study, we identified two patients with distinct clinical features and severity with two *de novo* variants in the *PPP3CA* gene, which codes for CaN catalytic subunit alpha, and investigated their effects exploiting a patient-derived LCLs.

Pt.1 fully satisfies the criteria for class 3 patients, according to the classification proposed by Panneerselvam et al.. in 2021 [6]: patients with a heterozygous mutation truncating PPP3CA RD domain, who display a loosely defined clinical spectrum that includes developmental delay, intellectual disability, abnormal EEG, early-onset epilepsy and brain anomalies. Pt.2 escapes the severe manifestation. This discrepancy could be due to the type of variant i.e. splicing variant and not falling in the coding region.

For Pt.1 the increase in isoform 2 expression could be explained by a compensatory mechanism implemented by the cell to offset the production of a nonfunctioning truncated protein; this mechanism is known to intervene to reverse inappropriate expression of certain genes to restore protein homeostasis [22] which found confirmation by WB analysis. The high mortality rate, on the other hand, may be given by the massive activation of the UPR due to the presence of truncated PPP3CA. Clarifying the molecular mechanism that underlies Pt.1 disorder requires defining whether the variant acts through a loss-of-function, haploinsufficiency mechanism, or through a gain-of-function/dominant-negative effect exerted by the truncated protein. Based on published biochemical data, the truncated products of Pt.1 *PPP3CA* variant are expected to be constitutively active and dysregulated as enzymes, due to the absence of the auto-inhibitory domain [23]. On the other hand, folding prediction algorithms model a misfolded structure for both isoform 1 and isoform 2 shortened proteins and their aberrant C-terminal tails (Fig. 2D-E’). This might prelude to their disposal by either the ubiquitin-proteasome pathway, or the autophagy-lysosome pathway (in case of aggregation). An unstable and degraded PPP3CA protein would point to a pathogenic effect descending from haploinsufficiency, which implies reduced wt protein amounts. WB analysis of Pt.1 LCL showed maintained levels of PPP3CA full-length products when compared with controls; it must be noted, however, that WB analysis was expected to reveal only the wt allele products (as the antibody available is directed against C-terminal amino acids, from residue 470) and doesn’t separate the closely migrating isoform 1 and 2 polypeptides. Specifically, WB tells nothing about the presence and relative abundance of mutated isoform 1 and 2 protein products.

Pt.1 *PPP3CA* mRNAs analysis, on the other hand, demonstrated a strong downregulation of isoform 1 and upregulation of isoform 2. Frameshift mutations resulting in PTC are targets of the nonsense-mediated decay (NMD) RNA surveillance system, but according to the “55 boundary rule”, PTC downstream of the last 55 nucleotides of the penultimate exon escape NMD [24], as in Pt.1, escape NMD. Releveling of mRNA isoforms could therefore reflect the activation of another compensatory mechanisms, maybe acting at the alternative splicing and/or mRNA stability levels, to selectively offset the mutated isoform 1 and enhance isoform 2; similar mechanisms have been shown to ensue for reversing inappropriate gene expression in numerous instances [22]. Intriguingly, isoform 1 is the prevailing splicing form in central nervous system.

Further studies are needed to determine whether *PPP3CA* mutation effect stem from an unbalanced isoforms ratio or the influence of stable truncated proteins. Regardless, disrupted CaN signaling likely imposes chronic cellular stress, explaining the reduced viability and elevated apoptotic death observed in Pt.1 LCL. Our data in particular highlight the upregulation of UPR target genes that drives the pro-apoptotic branch of UPR signaling [25], suggesting that constitutive activation of the UPR-apoptosis axis may play a significant role in Pt.1 pathogenesis.

As to Pt.2, his *PPP3CA* variant, though positionally at the boundary of the RD-encoding exon 13, is a splicing mutation that is expected to cause the skipping of exon 13. Experimental data indicate that wt protein levels are not significantly altered, and that mRNA, similarly, is only modestly reduced in isoform 2; this, again, can be regarded as a compensatory upregulation of wt allele transcription and splicing into isoform 1. The milder clinical phenotype of Pt.2 compared to all other patients reported in the literature with pathogenic variants in *PPP3CA*, would so be explained by the moderately imbalanced expression of the otherwise intact isoforms 1 and 2, in the absence of aberrant protein forms. On the other hand, the activation of cryptic SA sites could potentially generate small amounts of a truncated protein. Our attempts to identify by RT-PCR anomalous splicing forms in Pt.2 LCL failed (data not shown).

Intriguingly, also Pt.2 LCL displayed an upregulated UPR pathway, although disjoined from a robust activation of apoptosis. A chronically switched-on UPR might represent a common phenotype of PPP3CA-pathies; it would be interesting verifying this point in patients bearing bona fide loss-of-function CD domain mutations and gain-of-function AID domain mutations. Pt.2 mismatched phenotype is not a novelty in studies of genotype-phenotype correlation, especially in the context of neurodevelopmental diseases. With the advent of WES and WGS sequencing, the classical “one gene-one phenotype” pairwise relation has been subverted into “one gene-many phenotypes”, particularly in the field of neurodevelopmental diseases: the list of diseases in which not only distinct variants in the same gene, but even the same variant, can lead to different clinical phenotypes, is steadily growing, as exemplified by the Developmental and epileptic encephalopathies, Marfan Syndrome, Rubinstein–Taybi Syndrome [26–28] and many other disorders.

## Conclusions

Using WES-trio investigation we identified two *de novo* pathogenic variants in *PPP3CA*, a novel (in the coding region) and a splicing variant (in patient 2 described in Rosina et al.. 2024) [17] not only increasing the number of known variants reported for this gene, but also expanding its associated phenotypic spectrum throught the description of a patient with a splicing variant and a mild clinical manifestation, characterized by a moderate developmental delay, moderate speech delay, absence of visual, respiratory and feeding anomalies. This study clearly indicates the importance of a WES-trio analysis in presence of a nonspecific clinical picture and adds novel information for understanding the possible genotype-phenotype correlations and the pathogenetic mechanisms of *PPP3CA* variants, specifically with the finding of an upregulated UPR as a recurrent cell phenotype.

## Data Availability

The variant is available in the LOVD website (www.lovd.nl) with the variant ID #0000987643 and #0000987644.

## References

[1] Jiang H, Xiong F, Kong S, Ogawa T, Kobayashit M, Liu J. 0. Distinct tissue and cellular distribution of two major isoforms of calcineurin. Molecular Immunology. 1997, 34. 10.1016/s0161-5890(97)00054-0 9393969

[2] Creamer TP. Calcineurin. Cell Communication and Signaling. BioMed Central Ltd; 2020, 18. 10.1186/s12964-020-00636-4 PMC7456046 32859215

[3] Mertz P, Calcineurin. Form and Function. 2000. http://physrev.physiology.org 10.1152/physrev.2000.80.4.1483 11015619

[4] Eckel R, Szulc B, Walker MC, Kittler JT. Activation of calcineurin underlies altered trafficking of α2 subunit containing GABAA receptors during prolonged epileptiform activity. Neuropharmacology. 2015;88:82–90. 25245802 10.1016/j.neuropharm.2014.09.014 PMC4239296

[5] Wen Y, Fu P, Wu K, Si K, Xie Y, Dan W, et al. Inhibition of Calcineurin A by FK506 Suppresses Seizures and Reduces the Expression of GluN2B in Membrane Fraction. Neurochem Res. 2017;42(8):2154–66. 28299629 10.1007/s11064-017-2221-0

[6] Panneerselvam S, Wang J, Zhu W, Dai H, Pappas JG, Rabin R, et al. PPP3CA truncating variants clustered in the regulatory domain cause early-onset refractory epilepsy. Clin Genet. 2021;100(2):227–33. 33963760 10.1111/cge.13979 PMC11698261

[7] Chiocco MJ, Zhu X, Walther D, Pletnikova O, Troncoso JC, Uhl GR, et al. Fine mapping of calcineurin (PPP3CA) gene reveals novel alternative splicing patterns, association of 5′UTR trinucleotide repeat with addiction vulnerability, and differential isoform expression in Alzheimer’s Disease. Subst Use Misuse. 2010;45(11):1809–26. 20590401 10.3109/10826084.2010.482449 PMC3031160

[8] Mizuguchi T, Nakashima M, Kato M, Okamoto N, Kurahashi H, Ekhilevitch N, et al. Loss-of-function and gain-of-function mutations in PPP3CA cause two distinct disorders. Hum Mol Genet. 2018;27(8):1421–33. 29432562 10.1093/hmg/ddy052

[9] Myers CT, Stong N, Mountier EI, Helbig KL, Freytag S, Sullivan JE, et al. De Novo Mutations in PPP3CA Cause Severe Neurodevelopmental Disease with Seizures. Am J Hum Genet. 2017;101(4):516–24. 28942967 10.1016/j.ajhg.2017.08.013 PMC5630160

[10] Rydzanicz M, Wachowska M, Cook EC, Lisowski P, Kuźniewska B, Szymańska K, et al. Novel calcineurin A (PPP3CA) variant associated with epilepsy, constitutive enzyme activation and downregulation of protein expression. Eur J Hum Genet. 2019;27(1):61–9. 30254215 10.1038/s41431-018-0254-8 PMC6303256

[11] Yang S, Shen X, Kang Q, Kuang X, Ning Z, Liu S et al. Clinical and Genetic Study on a Chinese Patient with Infantile Onset Epileptic Encephalopathy carrying a PPP3CA Null Variant: A case report. BMC Pediatr. 2020;20(1). 10.1186/s12887-020-02213-7 PMC7320544 32593294

[12] Li J, Cao J. Case report: A novel PPP3CA truncating mutation within the regulatory domain causes severe developmental and epileptic encephalopathy in a Chinese patient. 10.3389/fneur.2022.889167 PMC9491239 36158964

[13] Baldo C, Viotti V, Maioli E, Mogni M, Castagnetta M, Cavani S et al. Galliera Genetic Bank: A DNA and Cell Line Biobank from Patients Affected by Genetic Diseases. Open J Bioresour. 2016;3.

[14] Quartier A, Courraud J, Thi Ha T, McGillivray G, Isidor B, Rose K, et al. Novel mutations in NLGN3 causing autism spectrum disorder and cognitive impairment. Hum Mutat. 2019;40(11):2021–32. 31184401 10.1002/humu.23836

[15] Hernandez-Segura A, Rubingh R, Demaria M. Identification of stable senescence-associated reference genes. Aging Cell. Blackwell Publishing Ltd; 2019, 18. 10.1111/acel.12911 PMC6413663 30710410

[16] Hruz T, Wyss M, Docquier M, Pfaffl MW, Masanetz S, Borghi L, et al. RefGenes: identification of reliable and condition specific reference genes for RT-qPCR data normalization. BMC Genomics. 2011;12:156. 10.1186/1471-2164-12-156 PMC3072958 21418615

[17] Rosina E, Pezzani L, Apuril E, Pezzoli L, Marchetti D, Bellini M et al. Comparison of first-tier whole-exome sequencing with a multi-step traditional approach for diagnosing paediatric outpatients: An Italian prospective study. Mol Genet Genomic Med. 2024;12(1). 10.1002/mgg3.2316 PMC10767581 38041506

[18] Ulengin-Talkish I, Cyert MS. A cellular atlas of calcineurin signaling. Biochim Biophys Acta Mol Cell Res. 2023;1870(1). 10.1016/j.bbamcr.2022.119366 PMC9948804 36191737

[19] Bollo M, Paredes RM, Holstein D, Zheleznova N, Camacho P, Lechleiter JD. Calcineurin interacts with PERK and dephosphorylates calnexin to relieve ER stress in mammals and frogs. PLoS ONE. 2010;5(8). 10.1371/journal.pone.0011925 PMC2916823 20700529

[20] Hetz C, Zhang K, Kaufman RJ. Mechanisms, regulation and functions of the unfolded protein response. Nature Reviews Molecular Cell Biology. Volume 21. Nature Research; 2020. pp. 421–38. 10.1038/s41580-020-0250-z PMC8867924 32457508

[21] Fribley A, Zhang K, Kaufman RJ. Regulation of apoptosis by the unfolded protein response. Methods Mol Biol. 2009;559:191–204. 19609758 10.1007/978-1-60327-017-5_14 PMC3684430

[22] Bravo-Estupiñan DM, Aguilar-Guerrero K, Quirós S, Acón MS, Marín-Müller C, Ibáñez-Hernández M, et al. Gene dosage compensation: Origins, criteria to identify compensated genes, and mechanisms including sensor loops as an emerging systems-level property in cancer. Cancer Medicine. John Wiley and Sons Inc; 2023;12:22130–55. 10.1002/cam4.6719 PMC10757140 37987212

[23] Li SJ, Wang J, Ma L, Lu C, Wang J, Wu JW, et al. Cooperative autoinhibition and multi-level activation mechanisms of calcineurin. Cell Res. 2016;26(3):336–49. 26794871 10.1038/cr.2016.14 PMC4783466

[24] Khajavi M, Inoue K, Lupski JR. Nonsense-mediated mRNA decay modulates clinical outcome of genetic disease. Eur J Hum Genet. 2006;14:1074–81. 16757948 10.1038/sj.ejhg.5201649

[25] Hu H, Tian M, Ding C, Yu S. The C/EBP homologous protein (CHOP) transcription factor functions in endoplasmic reticulum stress-induced apoptosis and microbial infection. Frontiers in Immunology . Frontiers Media S.A.; 2019, 10. 10.3389/fimmu.2018.03083 PMC6328441 30662442

[26] Guerrini R, Conti V, Mantegazza M, Balestrini S, Galanopoulou AS, Benfenati F, Developmental and epileptic encephalopathies: from genetic heterogeneity to phenotypic continuum. Physiological Reviews. American Physiological Society; 2023;103:433–513. 10.1152/physrev.00063.2021 PMC9576177 35951482

[27] Sun YH, Wu YL, Liao BY. Phenotypic heterogeneity in human genetic diseases: ultrasensitivity-mediated threshold effects as a unifying molecular mechanism. Journal of Biomedical Science. BioMed Central Ltd; 2023, 30. 10.1186/s12929-023-00959-7 PMC10388531 37525275

[28] Saettini F, Fazio G, Bonati MT, Moratto D, Massa V, Di Fede E, et al. Identical EP300 variant leading to Rubinstein–Taybi syndrome with different clinical and immunologic phenotype. Am J Med Genet A. 2022;188(7):2129–34. 35266289 10.1002/ajmg.a.62719

